# Capture–Recapture Method for Estimating Annual Incidence of Imported Dengue, France, 2007–2010

**DOI:** 10.3201/eid1911.120624

**Published:** 2013-11

**Authors:** Guy La Ruche, Dominique Dejour-Salamanca, Pascale Bernillon, Isabelle Leparc-Goffart, Martine Ledrans, Alexis Armengaud, Monique Debruyne, Gérard-Antoine Denoyel, Ségolène Brichler, Laetitia Ninove, Philippe Desprès, Marc Gastellu-Etchegorry

**Affiliations:** Institut de Veille Sanitaire, Saint-Maurice, France (G. La Ruche, D. Dejour-Salamanca, P. Bernillon, M. Gastellu-Etchegorry);; Institut de Recherche Biomédicale des Armées, Marseille, France (I. Leparc-Goffart); Cellule de l'Institut de Veille Sanitaire aux Antilles-Guyane, Fort-de-France, Martinique, France (M. Ledrans);; Cellule de l'Institut de Veille Sanitaire Sud, Marseille (A. Armengaud);; Cerba Laboratory, Saint-Ouen l’Aumone, France (M. Debruyne);; Biomnis Laboratory, Lyon, France (G.-A. Denoyel);; Avicenne University Hospital, Bobigny, France (S. Brichler);; Institut Hospitalo-Universitaire Méditerranée-Infection, Marseille (L. Ninove);; Institut Pasteur National Reference Laboratory for Arboviruses, Paris, France (P. Desprès)

**Keywords:** dengue, viruses, Aedes mosquitoes, incidence, population surveillance, disease notification, epidemiologic methods, France, travel medicine

## Abstract

Imported dengue cases pose the public health risk for local circulation in European areas, especially southeast France, where the *Aedes* mosquito is established. Using a capture–recapture method with Chao’s estimator, we estimated the annual incidence of dengue fever and the completeness of existing mandatory notification and laboratory network surveillance systems. During 2007–2010, >8,300 cases with laboratory evidence of recent dengue infection were diagnosed. Of these cases, 4,500 occurred in 2010, coinciding with intense epidemics in the French West Indies. Over this 4-year period, 327 cases occurred in southeast France during the vector activity period. Of these, 234 cases occurred in 2010, most of them potentially viremic. Completeness of the mandatory notification and laboratory network systems were ≈10% and 40%, respectively, but higher in southeast areas during May–November (32% and 69%, respectively). Dengue surveillance systems in France provide complementary information that is essential to the implementation of control measures.

Dengue fever, caused by 4 virus serotypes, is the most common mosquito-borne viral disease in the world: an estimated 50 million cases occur annually (*1*). During the past 50 years, incidence has increased 30-fold with increasing geographic expansion (*1*). In Europe, imported cases among travelers returning from endemic or epidemic countries have been reported frequently during recent years. Considering the risk for a local cycle of transmission and subsequent epidemic, imported dengue cases pose a potential public health problem in European areas where a competent vector is established. Since 2004, the *Aedes albopictus* mosquito has been established in southeast France (*2*,*3*).

During 2010, the first 2 known cases of autochthonous dengue fever were diagnosed in persons in metropolitan France (*4*), which comprises continental France and the island of Corsica, located southeast of mainland France (Figure 1). Two cases were also reported in Croatia during 2010 (*5*), demonstrating that local transmission in continental Europe is a reality. Accordingly, in the context of implementing appropriate public health measures, dengue surveillance systems should be able to estimate the incidence of imported symptomatic cases, describe their geographic distribution in areas already or potentially colonized by the competent vector, and identify the countries where infection occurred. Using a capture–recapture method, we estimate the annual incidence of imported dengue cases and the completeness of the existing surveillance systems in metropolitan France during 2007–2010.

**Figure 1:**
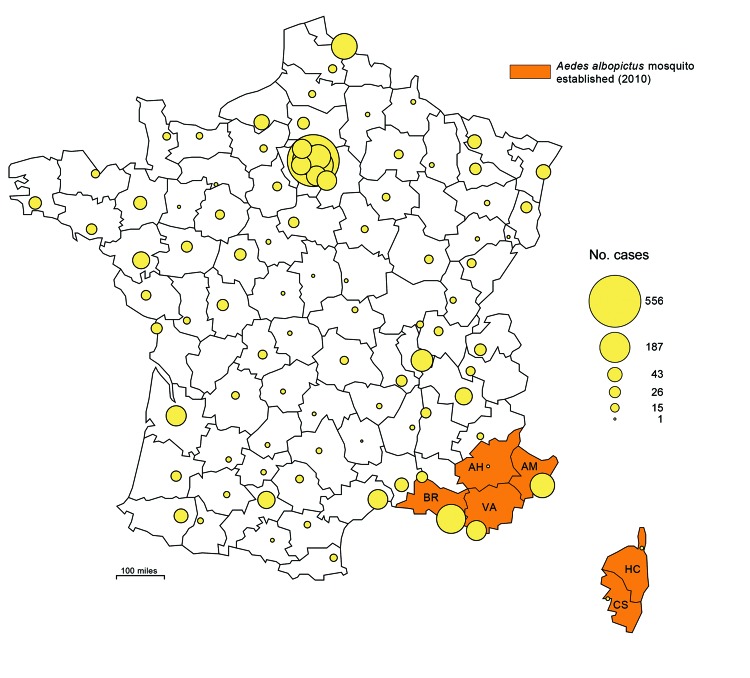
Geographic distribution of dengue cases in the departments (administrative districts) of metropolitan France, 2007–2010, and departments where the vector was established in 2010. Circles in outlined departments represent dengue cases reported by 3 surveillance systems. AH, Alpes-de-Haute-Provence; AM, Alpes-Maritimes ; BR, Bouches-du-Rhône; CS, Corse-du-Sud; HC, Haute-Corse; VA, Var. (Map made with Philcarto, http://philcarto.free.fr/)

## Methods

### Dengue Surveillance Systems

As of 2010, the *Ae. albopictus* mosquito was designated as permanently established in 6 southeast departments (administrative districts) in metropolitan France as follows: Alpes-Maritimes (2004), the 2 departments that comprise Corsica, Haute-Corse (2006) and Corse-du-Sud (2007), Var (2007), Bouches-du-Rhône (2009), and Alpes-de-Haute-Provence (2010). Entomological surveillance, based on data from the monitoring of ovitraps (*3*), enabled information on distribution of this mosquito to be updated within a few weeks. In addition to entomologic surveillance, since 2006, health authorities in France have implemented 3 complementary epidemiologic surveillance systems to identify new dengue and chikungunya infections: a notifiable diseases system that relies on mandatory notification, a laboratory-based surveillance system that operates at the national level, and an enhanced surveillance system, activated each year during May–November in the departments where the *Ae. albopictus* mosquito is established (*3*).

The notifiable diseases system requires mandatory notification by practitioners and biologists to collect clinical and biological information about recent symptomatic dengue cases. Notifiable cases are defined by recent fever (within 7 days of medical examination) associated with pain (headache, arthralgia, myalgia, low back pain, or retro-orbital pain) and positive test results for 1 of the following biologic results indicative of dengue infection: reverse transcription PCR (RT-PCR), nonstructural protein 1 [NS1] antigenic test, or IgM serologic analysis. Notification is centralized by the French Institute for Public Health Surveillance for the purpose of epidemiologic analysis. It has been shown that mandatory notification systems lack completeness (of unknown magnitude) and representativeness, and overrepresent hospitalized case-patients (*6*), leading to unequal probability of being included in a sample (catchability) (*7*) of dengue cases.

The laboratory-based national surveillance system is a voluntary network that comprises 6 specialized laboratories that monitor the trends of dengue diagnosis (*8*). Dengue cases are defined by positive RT-PCR, NS1 or IgM serologic test results, regardless of clinical signs. These biologic tests are only to be prescribed when a patient has suggestive symptoms. They are reported weekly to the French Institute for Public Health Surveillance. A survey during 2006 showed that this laboratory network aggregated ≈85% of the biologic diagnoses of dengue performed in metropolitan France (*9*).

The enhanced surveillance system is implemented in the departments where the vector is established, during its period of activity from May 1–November 30 each year. Unlike mandatory notification, the basis of enhanced surveillance is the immediate reporting of all clinically suspected cases of dengue fever by practitioners to the regional health authorities. This facilitates accelerated biologic confirmation by the national reference laboratory for arboviruses and, when appropriate, the rapid implementation of local control measures such as perifocal vector control activities and active case finding (*3*,*4*).

The 3 surveillance systems are obviously interconnected. For example, during the period of vector activity, dengue cases obtained from mandatory notification or from the laboratory network are immediately reported by the French Institute for Public Health Surveillance to the regional officers supervising the enhanced surveillance system.

A person with an imported case was defined as having traveled in an area where the dengue virus circulates within the previous 15 days before the onset of symptoms. As no substantial local transmission cycle occurred during the study period, all cases without patient information on travel history were considered imported cases.

### Strategy for Statistical Analysis

We used the capture–recapture method to estimate the incidence of imported dengue cases in metropolitan France during 2007–2010. By identifying common cases from several systems, this method provides an estimate of the number of cases not captured by any data sources. Consequently, the total number of cases and the capture probabilities of cases within each source can be estimated. To identify common cases, we checked the 3 data sources to find the patient’s date of birth, sex, and postal code of residence or of the laboratory where blood samples were collected, and the date of blood sampling. We faced 2 main obstacles to using the capture–recapture method. First, 1 of the 3 data sources, the enhanced surveillance system, operates in a limited area during 7 months each year. This obstacle restricted the possibility of comparing the 3 sources for the analysis. Second, the functional interrelationships between the 3 dengue surveillance systems appear to be limitations to the use of the standard 2-source capture–recapture methods and need to be quantified. We therefore conducted the analysis in 2 steps.

**Figure 2:**
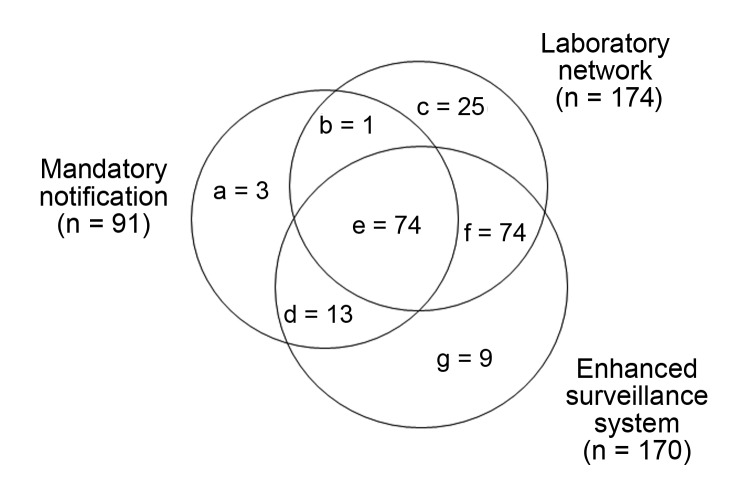
Distribution of 199 dengue cases among 3 surveillance systems, southeast France, May 1–November 30, 2010.

First, dependencies between sources were statistically evaluated following suggestions of Wittes et al. (*10*,*11*). The odds ratio implemented with the capture–recapture technique, developed by Wittes et al., is an estimate of the increased probability of a dengue case being reported in a first source when it is also reported in a second source. To investigate the relationship between these sources, the analysis is restricted to cases identified by a third source. The dependence analysis of the sources was restricted to the year 2010 because of an insufficient number of dengue cases before this date. Figure 2 shows the distribution among the 3 surveillance systems of the 199 biologically confirmed dengue cases detected during May 1–November 30, 2010, in the 6 departments of southeast France where the mosquito was established. Table 1 details the calculation of statistical interdependence of the 3 sources. The enhanced surveillance system is highly dependent on both the mandatory notification and the laboratory network. In contrast, mandatory notification and the laboratory network are systems that do not seem to be substantially interdependent; accordingly, we retained only these 2 reporting mechanisms to estimate the annual dengue incidence.

**Table 1:** Interdependence of laboratory network, mandatory notification, and enhanced surveillance systems in the detection of dengue cases, southeast France, May 1–November 30, 2010

	Laboratory network		Odds ratio (95% CI*)	Enhanced surveillance		Odds ratio (95% Cl*)
Surveillance system	Included	Not included		Included	Not included	
Mandatory notification			0.7 (0.3–1.9)			25.0 (3.8–1040.0)
Included	74	13		74	1	
Not included	74	9		74	25	
Enhanced surveillance			17.1 (1.2–907.9)			
Included	74	13				
Not included	1	3				
*95% CI calculated using exact confidence limits.						

Second, the capture–recapture method was applied to these 2 national-level sources by using 2 estimators of population size: the Chapman-Seber (CS) and the Chao estimates. The CS estimator (*12*,*13*) is a commonly used formula and is considered. This formula uses the hypotheses of independence between sources and equal catchability by each source. Instead, it has been shown recently that Chao’s estimator, as formulated by Brittain, is less biased than the CS estimator when there is dependence between sources or unequal catchability of cases (*14*). Therefore, in this study, we gave priority to the results obtained by using Chao’s estimator.

For both estimators, the 95% CIs associated with population size estimates were calculated with the log-transformation suggested by Burnham and used by Chao (*15*,*16*). The corresponding completeness values and their 95% CIs, obtained by using Monte-Carlo simulations, were calculated for mandatory notification, for the laboratory network, and for the combined surveillance systems.

Stratification was made for geographic areas where the *Ae. albopictus* mosquito was established versus other areas and by period of the year (vector activity period versus the rest of the year) to take into account and reduce the potential inequality in catchability. The total variance was calculated by adding the variance of each stratum. To acquire an understanding of the general shape of the curve of monthly number of cases and to compare it with that obtained for the French West Indies, we used the CS estimator with stratification according to geographic area, as many zero values precluded using the Chao estimator.

## Results

During 2007–2010 in metropolitan France, 773 cases of dengue were reported by mandatory notification, 3,192 by the laboratory network, and 180 by the enhanced surveillance system. A total of 3,432 distinct cases were reported by the 3 sources, distributed throughout the French departments (Figure 1). Of these cases, 3,423 were reported by 1 of the 2 national level sources: mandatory notification or the laboratory network. The male:female sex ratio of the patients was 0.99:1, and the median age of the patients was 41 years. Positive biologic diagnosis of dengue infection was made by IgM serology (83%) and by an antigenic method (17%, RT-PCR: 13% and NS1 test: 4%). For 1,204 cases (35%), anamnestic and biologic information was sufficient to determine the viremic status of the patients in metropolitan France. Among them, 48.5% were biologically viremic (positive RT-PCR or NS1 test), 47.8% were potentially viremic (the delay between the onset of symptoms and their subsequent return journey to France was <8 days) and 3.7% had data which were not compatible with the viremic stage (viremia delay had exceeded 7 days). Viremia usually occurs between the day before the onset of symptoms and the seventh day after.

The following additional clinical and biological information was available for 718 of the 773 cases of dengue reported by the mandatory notification system. Of the 718 patients, hemorrhagic symptoms were reported for 134 (19%) during the 4-year period. Eight (1%) of these had severe hemorrhagic symptoms (defined at the time of the study as tourniquet test, mucocutaneous bleeding, bleeding from puncture sites, or visceral bleeding). Of the patients for whom specific information was available, 51% (330/652) had thrombocytopenia (platelets ≤100,000/mL) and 47% (323/683) were hospitalized.

### Annual Incidence of Dengue Cases and Completeness of Surveillance Systems

Table 2 shows the total number of imported dengue cases in France during the study period: the annual number of cases estimated from mandatory notification and the laboratory network systems after stratification by area where the *Ae. albopictus* mosquito was established, and by the vector activity period. Using the Chao estimator, we calculated the global number of dengue cases to be 8,374 (95% CI 7,543–9,371) during the 4-year period; the estimated annual incidences were 2,109 in 2007, 751 in 2008, 1,057 in 2009, and 4,456 in 2010 (Table 2). These figures were much lower when we used the CS estimator: the global number of dengue cases was estimated at 4,818 during the 4-year period, 1.7 × lower than that estimated by using the Chao estimator (Table 3).

**Table 2:** Annual number of dengue cases estimated from mandatory notification and laboratory network surveillance systems by using Chao estimator, stratified according to geographic area and period of the year, metropolitan France, 2007–2010*

	No. observed cases				Chao estimator			
	System							
Year, region, period	MN	LN	Common cases		Estimated no. cases (95% CI)	Completeness of MN, % (95% CI)		Completeness of LN, % (95% CI)
2007								
Southeast, May–Nov	4	21	4		39 (26–87)		10.2 (2.4–17.2)	53.8 (12.6–90.4)
Other areas, remainder of year	52	365	21		2,070 (1,444–3,071)		2.5 (1.6–3.9)	17.6 (10.9–27.1)
Total	56	386	25		2,109 (1,481–3,109)		2.7 (1.6–4.0)	18.3 (11.3–27.5)
2008								
Southeast, May–Nov	6	11	4		18 (14–37)		33.2 (7.6–44.8)	60.9 (13.9–82.2)
Other areas, remainder of year	58	293	42		733 (595–938)		7.9 (5.9–10.0)	40.0 (30.0–50.6)
Total	64	304	46		751 (613–956)		8.5 (6.3–10.6)	40.5 (29.8–50.5)
2009								
Southeast, May–Nov	5	19	4		36 (24–79)		13.9 (3.1–22.9)	52.8 (11.6–87.2)
Other areas, remainder of year	63	331	38		1,021 (806–1,339)		6.2 (4.5–8.1)	32.4 (23.7–42.4)
Total	68	350	42		1,057 (841–1,375)		6.4 (4.6–8.2)	33.1 (23.6–42.4)
2010								
Southeast, May–Nov	91	174	75		234 (216–264)		38.9 (33.2–42.7)	74.3 (63.6–81.6)
Other areas, remainder of year	494	1,951	354		4,222 (3,932–4,558)		11.7 (10.8–12.6)	46.2 (42.7–49.7)
Total	585	2,125	429		4,456 (4,164–4,792)		13.1 (12.2–14.1)	47.7 (44.2–51.1)
2007–2010								
Southeast, May–Nov	106	225	87		327 (294–382)		32.4 (17.2–35.7)	68.8 (36.5–75.8)
Other areas, remainder of year	667	2,940	455		8,047 (7,217–9,045)		8.3 (7.1–9.2)	36.5 (31.1–40.7)
Total	773	3,165	542		8,374 (7,543–9,371)		9.2 (8.2–10.7)	37.8 (33.5–43.8)

*Information on geographic area or period of the year was not available for 27 cases. MN, mandatory notification; LN, laboratory network.

**Table 3:** Annual number of dengue cases estimated from MN and LN surveillance systems by using Chapman-Seber estimator, stratified according to geographic area and period of the year, metropolitan France, 2007–2010*

	No. observed cases					Chapman-Seber estimator	
Year, region, period	System		Common cases		Estimated no. cases (95% CI)	Completeness of MN, % (95% CI)	Completeness of LN, % (95% CI)
	MN	LN					
2007							
Southeast, May–Nov	4	21	4		21†	19.0†	100†
Other areas, remainder of year	52	365	21		881 (678–1,228)	5.9 (4.2–7.7)	41.4 (29.7–53.8)
Total	56	386	25		902 (699–1,249)	6.2 (4.5–8.0)	42.8 (30.9.7–55.2)
2008							
Southeast, May–Nov	6	11	4		16 (14–27)	38.0 (22.0–44.4)	69.6 (40.3–81.2)
Other areas, remainder of year	58	293	42		402 (360–479)	14.4 (12.1–16.1)	72.8 (61.2–81.3)
Total	64	304	46		418 (376–495)	15.3 (12.9–17.0)	72.7 (61.2–80.7)
2009							
Southeast, May–Nov	5	19	4		23 (20–38)	21.7 (13.1–24.4)	82.6 (52.3–97.6)
Other areas, remainder of year	63	331	38		544 (468–671)	11.6 (9.4–13.5)	60.9 (49.3–70.1)
Total	68	350	42		567 (490–694)	12.0 (9.8–13.8)	61.7 (50.3–71.2)
2010							
Southeast, May–Nov	91	174	75		211 (200–232)	43.2 (39.3–45.4)	82.5 (75.2–86.8)
Other areas, remainder of year	494	1,951	354		2,721 (2,599–2,872)	18.2 (17.2–19.0)	71.7 (67.9–75.1)
Total	585	2,125	429		2,932 (2,809–3,083)	20.0 (19.0–20.8)	72.5 (68.9–75.6)
2007–2010							
Southeast, May–Nov	106	225	87		271 (258–294)	39.2 (35.4–40.8)	83.1 (75.1–86.7)
Other areas, remainder of year	667	2,940	455		4,548 (4,262–4,907)	14.7 (13.5–15.5)	64.6 (59.3–68.5)
Total	773	3,165	542		4,818 (4,532–5,178)	16.0 (14.8–16.9)	65.7 (60.5–69.3)
*Information on geographic area or period of the year was not available for 27 cases; MN, mandatory notification; LN, laboratory network. †95% CI not presented because of null variances.							

During the period of vector activity (May–November), the estimated number of dengue cases was 39 (95% CI 26–87) for 2007 and 18 (95% CI 14–37) for 2008 in the 4 departments where the *Ae. albopictus* mosquito was established at that time; the estimated number was 36 (95% CI 24–79) for 2009 and 234 (95% CI 216–264) for 2010, when a fifth and sixth department, respectively, became affected (Table 2). Dengue cases which occur in these areas during the vector activity period have potential public health implications, given the risk for local transmission by viremic persons.

During 2007–2010, among the 327 estimated dengue cases that occurred in the areas where the vector was established and during its period of activity, 83 (25%) were not detected by either of the 2 national level surveillance systems. The enhanced surveillance system detected 85% of the 199 observed cases (Figure 2) and 73% of the 234 estimated cases in 2010. This system helped to catch a few of the additional cases in 2010 (9 among 199 cases, 4.5%) which were not detected by either the mandatory notification or laboratory network surveillance systems.

Finally, among the 199 patients in whom dengue was detected in southeast France during May–November 2010, 93 (47%) had viremic dengue infections, 64 (32%) were potentially viremic, and 13 (7%) were not viremic. There was not sufficient information for the remaining 29 patients (15%) to enable classification. Among the biologically and potentially viremic patients, the mean estimated duration of viremia while they were in metropolitan France was 6 days.

The completeness of the 2 surveillance systems differed greatly; completeness was much higher for the laboratory network (Table 2). Using the Chao estimator, we estimated the completeness at 3% in 2007 and 13% in 2010 for the mandatory notification surveillance system (9.2% for the 4-year period). We estimated completeness to be an average of ≈4 times higher for the laboratory network: 18% in 2007 and 48% in 2010 (37.8% for the 4-year period). Furthermore, for both surveillance systems, completeness was much higher in areas where the competent vector was established (20.3% for mandatory notification and 57.3% for the laboratory network over the 4-year period) than in other areas (8.6% and 37.1%, respectively), and also much higher during the vector activity period (12.5% and 44.2%, respectively) than during the rest of the year (3.4% and 25.3%, respectively) (Table 4). For the 4-year period, these figures were 32% and 69%, respectively, in *Aedes* spp.-infested areas during May–November (Table 2). The combination of the 2 surveillance systems increased the completeness compared with the use of the laboratory network alone, but this increase was limited: ≈2% to 4% (Table 4). Globally, the combined completeness was ≈40% for the 4-year period.

**Table 4:** Number of dengue cases estimated from mandatory notification and laboratory network surveillance systems with Chao estimator after stratification according to geographic area and period of the year, metropolitan France, in 2010 and for 2007–2010*

Stratification type, Y	No. observed cases			Estimated total no. cases (95% CI)	Completeness of MN, % (95% CI)	Completeness of LN, % (95% CI)	Combined completeness, % (95% CI)
	With MN	With LN	Common cases				
Geographic stratification 2010							
Southeast	97	229	80	332 (302–378)	29.2 (25.0–32.5)	69.0 (59.0–76.8)	74.1 (63.4–82.5)
Other areas	488	1,896	349	4,071 (3,791–4,396)	12.0 (11.1–12.9)	46.6 (43.1–50.0)	50.0 (46.2–53.8)
Total	585	2,125	429	4,403 (4,120–4,730)	13.3 (12.3–14.2)	48.3 (44.8–51.6)	51.8 (48.1–55.4)
2007–2010							
Southeast	113	320	93	558 (475–691)	20.3 (16.3–23.8)	57.3 (46.3–67.3)	60.9 (49.2–71.5)
Other areas	660	2,847	449	7,684 (6,896–8,632)	8.6 (7.6–9.6)	37.1 (32.9–41.2)	39.8 (35.3–44.3)
Total	773	3,167	542	8,242 (7,446–9,194)	9.4 (8.4–10.4)	38.4 (34.4–42.5)	41.2 (37.0–45.6)
Period stratification							
2010							
May–Nov†	524	1,694	386	3,186 (2,994–3,410)	16.4 (15.3–17.5)	53.2 (49.6–56.6)	57.5 (53.6–61.3)
Remainder of year	61	431	43	1,407 (1,118–1,821)	4.3 (3.2–5.6)	30.6 (22.9–39.6)	31.9 (23.8–41.2)
Total	585	2,125	429	4,594 (4,223–5,034)	12.7 (11.4–13.9)	46.3 (41.5–50.4)	49.7 (44.6–54.1)
2007–2010							
May–Nov	656	2,325	468	5,263 (4,764–5,873)	12.5 (11.4–13.9)	44.2 (39.6–48.8)	47.7 (42.8–52.8)
Remainder of year	117	865	74	3,416 (2,761–4,303)	3.4 (2.7–4.2)	25.3 (20.1–31.3)	26.6 (21.1–32.9)
Total	773	3,190	542	8,679 (7,817–9,709)	8.9 (8.0–9.9)	36.8 (32.9–40.8)	39.4 (35.2–43.8)
*MN, mandatory notification; LN, laboratory network; information on geographic area or period of the year was not available for 27 cases. Southeast includes the departments (administrative regions) where the competent vector, the *Aedes albopictus* mosquito, was established. †May–Nov is the period of activity of *Ae. albopictus* mosquitoes in metropolitan France.							

### Geographic Area of Acquisition of Dengue Infection and Influence of Epidemics in French West Indies

Information on the country of acquisition of dengue infection was available for 1,335 patients (this information was available for mandatory notification and for 3 of the 6 laboratories). Dengue was acquired mainly from 2 geographic areas: the Caribbean and Southeast Asia, which represented 61% and 17% of cases, respectively, over the 4-year period (Table 5). In the Caribbean, the most frequent areas of acquisition (59% of all reported cases) were the French West Indies including Martinique, Guadeloupe, Saint-Barthelemy, and Saint-Martin. In Southeast Asia, dengue fever was primarily acquired in Thailand (7% of all cases) and Indonesia (5%).

**Table 5:** Geographic area of acquisition (%) among patients* with dengue infection in metropolitan France, 2007–2010

Region†	2007 (n = 109)	2008 (n = 119)	2009 (n = 151)	2010 (n = 956)	2007–2010 (n = 1,335)	
Africa					
Central	2.8	0.8	0.7	0.6	0.8	
East	6.4	1.7	0.7	2.6	2.6	
West	4.6	21.8	11.9	1.8	4.9	
Americas						
Caribbean	42.2	22.7	19.9	75.0	61.4	
Central America	3.7	3.4	4.0	0.4	1.3	
South America	8.3	5.9	13.9	2.8	4.8	
Asia						
Central	6.4	7.6	8.6	3.9	4.9	
Southeast	20.2	28.6	32.5	12.8	17.0	
South Pacific						
Polynesia	5.5	7.6	6.0	0.0	1.8	
Other areas in the South Pacific	0.0	0.0	2.0	0.1	0.3	

*Information was available for mandatory notification and for 3 of the 6 laboratories involved in the surveillance network. †Geographic regions and sub-regions: United Nations Statistics Division (http://unstats.un.org/unsd/methods/m49/m49regin.htm).

**Figure 3:**
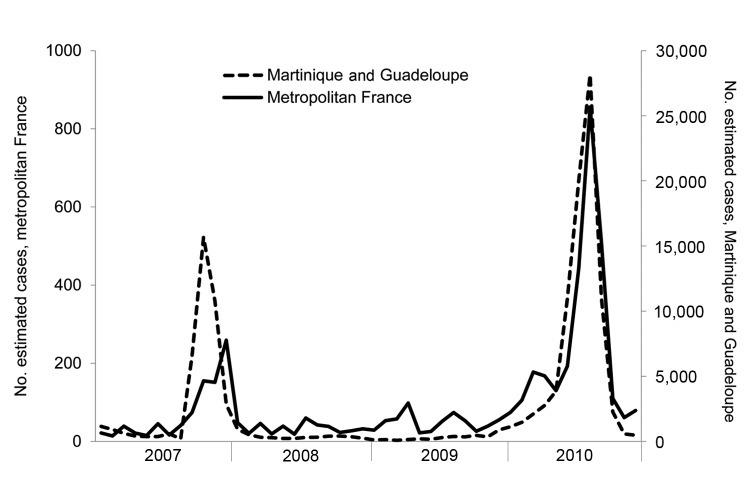
Estimated monthly numbers of dengue cases in metropolitan France* and in the French West Indies†, 2007–2010. *In metropolitan France, which comprises the mainland and the island of Corsica, biologically diagnosed cases of dengue were estimated by using the capture–recapture method with data from 2 sources: a mandatory notification system and a laboratory network; the Chapman-Seber estimator was used. Data were stratified according to geographic area. †On the French West Indies islands Martinique and Guadeloupe, cases were estimated from clinically suspected cases within a sentinel network of general practitioners on each island.

In 2007 and 2010, respectively, 34% (37/109) and 71% (682/956) of all cases imported to France were acquired in Guadeloupe (19% in 2007, 41% in 2010) and in Martinique (14% in 2007, 30% in 2010); on each of the 2 islands, dengue epidemics affected nearly 20,000 persons in 2007 and 40,000 in 2010 (*17*,*18*). Figure 3 shows the monthly number of dengue cases estimated during 2007–2010 in metropolitan France (estimation from the 2 national sources by using capture–recapture with CS estimator) and in Guadeloupe and Martinique (estimation by regional health authorities from clinically suspected cases within the sentinel network of physicians). The curves roughly overlap, especially during epidemics in the French West Indies. More precisely, the peaks of imported cases in metropolitan France coincide with those of epidemics which occurred in the French West Indies, particularly for the year 2010.

## Discussion

In this study, we estimated that >8,300 cases with laboratory evidence of recent dengue infection were imported into metropolitan France during 2007–2010. Approximately 4,500 of them occurred in 2010; this high number was mainly attributed to epidemics of unusually intense and long duration in the French West Indies (*19*). A correlation between a substantial number of imported cases of disease in metropolitan France and an intense epidemic in French overseas territories was observed with the dengue epidemic in the French West Indies in 2001 (*20*) and with the chikungunya epidemic on Reunion Island in 2006 (*21*). A similar contemporary association was observed in Germany, where an increase in imported dengue cases during 2002 was directly linked to an epidemic in Brazil (*22*).

An estimated 230 cases occurred during May–November 2010 in the 6 southeast departments of France where the *Ae. albopictus* mosquito was established, and >90% of the infected persons may have been viremic. The increase in the number of imported cases in southeast France and the high vector density in some urban areas were major factors in the emergence of a local transmission cycle. Two cases of autochthonous dengue fever were reported in the Alpes-Maritimes Department in September 2010 (*4*).

The capture–recapture method which we applied to estimate the incidence of imported dengue cases is widely used in epidemiologic surveillance studies when several sources of data are available (*23*). Several assumptions must be checked when using this method (*23*,*24*) to avoid biases. Among these, independence between sources and equal catchability of cases by each source, which are interdependent concepts (*7*), are of great importance. When log-linear modeling is used, the availability of at least 3 sources ensures that the dependence between sources and the unequal catchability when estimating the true number of cases (*24*,*25*) can be taken into account. When only 2 sources of data are available, as is the case for dengue surveillance in metropolitan France at the national level, statistical alternatives are necessary. In our study, the alternative used was the Chao estimator which relaxes the assumption that sources are independent, and provides more reliable estimates when the differences between the identifying probabilities of the 2 sources are large (*26*,*27*). In contrast, with increasing dependencies between sources, the commonly used CS estimator underestimates the true number of cases (*14*). This underestimation may explain the differences we found between the 2 estimates.

Furthermore, we reduced unequal catchability by stratifying the results by period of year and geographic area. Other factors associated with unequal catchability should be taken into consideration, but it was not possible to do so comprehensively in our study. In particular, patients with severe disease may have had a higher probability of being captured by surveillance systems, which would lead to an underestimation of dengue infections (*28*). Conversely, the risk for false positives when using IgM detection for dengue diagnosis may have led to an overestimation of this number.

As in other countries, the dengue surveillance systems in France aim to identify symptomatic patients. The proportion of asymptomatic or mildly symptomatic dengue infections fluctuates within endemo-epidemic countries (*29*) and equals ≈75% (*29*,*30*) of all dengue infections. Our estimate, based on symptomatic cases, must therefore be multiplied by 4 to provide a total number of imported dengue cases in France: ≈33,000 cases for the 2007–2010 period, including 1,300 cases in the area where the competent vector was established and during its period of activity. However, the role of asymptomatic dengue cases in the transmission of the virus to the competent vector is still not well known. In other words, viremia could be lower and shorter in duration in asymptomatic persons than in their symptomatic counterparts (*31*) and it is not certain that viremia of asymptomatic or mildly symptomatic persons is sufficient to be infective. From a public health point of view, the routine detection of asymptomatic infections returning from abroad is inconceivable.

## Conclusions

Completeness of the 2 national-level surveillance systems differed greatly: the variation was ≈10% for mandatory notification and ≈40% for the laboratory network. For both surveillance systems, completeness was much higher in the area where the competent vector was established, and during the vector’s period of activity; these factors represent the main target of the surveillance system. Although this finding is comforting in terms of ensuring the implementation of measures aimed at limiting the risk for a local cycle transmission, additional efforts should be made to further increase completeness. The low completeness level of mandatory notification brings up the question of its real usefulness for the early detection of cases and the implementation of control measures, especially because it only marginally improves the completeness of the laboratory network. However, the mandatory notification system in France does monitor the trends of imported cases, including those from countries where no dengue surveillance systems are in place, as is the case in most countries in western Africa (*32*). Furthermore, the mandatory notification system collects additional clinical information (symptoms, severity) which can be analyzed according to the serotype. This system is especially useful for surveillance of severe cases. 

The laboratory network system is used for the monitoring of spatial and temporal trends of dengue fever among travelers, and the assessment of the risk for importation into metropolitan France, including areas where the vector is already established or is likely to become established. In our study, the observed geographic origin of imported cases can probably be simultaneously explained by the global epidemiology of dengue, traveler flows to France, and the practices used to request laboratory diagnosis. Travelers returning from Antilles during intense dengue epidemics were among those who introduced the greatest number of cases. Such a situation may recur more frequently in the future as the epidemiology of dengue continues to become hyperendemic in these territories (*19*). Furthermore, the number of imported cases may increase because of the expansion of dengue and the increase of travelers.

The third system, enhanced surveillance, completes the framework. It contributes to the detection of a few additional cases that were not detected by the other 2 surveillance systems. The enhanced surveillance system leads to faster detection of the great majority of cases in the areas where *Ae. albopictus* mosquitoes were found compared with the 2 other surveillance systems. It results in the immediate implementation of local control measures. Moreover, this local enhanced surveillance supports the annual mobilization of professionals during the vector activity period, including health stakeholders in the areas where the vector is expanding.
